# Monthly Summary

**Published:** 1872-09

**Authors:** 


### MONTHLY SUMMARY.

*Human Ruminants.*—At Fuerth, near Nuremberg, there have been observed three ruminants, all of the male sex. The one was twenty-one years old, and had lost his father a long time. The mother suffered with pyrosis He had this anomalous state from his tenth year. The beginning was that he drank bad water in a wood, vomited it several times, and had for a long time afterwards nausea. In his last school years the rumination was going on. In another hospital he has been treated by emetics. The vomiting proceeded easily, but the rumination did not disappear. The other functions are all in order, except the defecation, which is somewhat inactive. Food and liquids easily reach the stomach. Not very long after the food the substances again mount to the throat. Then they return to the mouth first that which he had eaten the last, without pain or nausea. Only when he is quiet and not excited he is able to retain the food in the stomach, and some kinds of it only, beans and lentils, return most of all; then rice and barley; less frequently farinaceous, more or less meat (sometimes, but after three hours;) and he very seldom drinks. If the food comes soon after dinner. then the regurgitation is agreeable to him, and he masticates it with pleasure. For the beginning the mass is more thick, then thinner; it becomes, by and by, bitter and acid, then he cannot retain it, and rejects it, but without an effort to vomit. The tongue is always covered, and changes the epithelium. By night he never ruminates, by day he can as he wishes make the food return from the stomach. Dr. Heiling, of Nuremberg, wrote a pamphlet on rumination. He says that Darwin knew a man who swallowed red and white gooseberries, and then at the will of the spectators, caused the white or red ones to return separately. Very often hypertrophy or extensions of the stomach in the form of pouches, were the reason, of this rumination.—*Medical Press and Circular.*

---

*Cement for Glass and Porcelain.*—The very best of this kind, which will withstand moisture and even a moderate degree of heat, is the one proposed by Berzelius, and for the last year or so sold by enterprising venders in the streets of New York. It is nothing but the curd of skimmed milk (obtained by the addition of vinegar or rennet), beaten to a paste with quicklime in fine powder. A better quality than this is obtained in the following way: Add vinegar one-half pint, to skimmed milk one-half pint; mix the curd with the white of five eggs, well beaten, and enough quicklime to form a paste.—*Druggists Circular.*

*Iodized Cotton.*—M. Mehu, pharmacien at the Necker Hospital, describes his mode of impregnating cotton with iodine. The iodine is first reduced to an extremely fine powder by trituration in a porcelain mortar, its pulverization being greatly facilitated by adding, from time to time, some drops of ether. Very fine and quite dry carded cotton should be chosen, its weight being ten times greater than that of the iodine. This is introduced in small portions, with the portions of iodine, into a flask capable of containing a litre, and having a wide mouth. Into such a flask from twenty to twenty-five grammes of the cotton are sufficient to be introduced, in order to secure its thorough permeation by the iodine. The flask is placed in a sand-bath, only slightly corked, until the heated air has escaped, when it should be closely stopped. The iodine disengaged now thoroughly permeates the cotton, and when this has attained the color of roasted coffee the process is finished. As much as ten per cent. of iodine may be thus combined, but for all ordinary purposes 5 per cent. suffices. The cotton may be prepared, also, in a boiling water bath. To preserve it for use it must be kept in a bottle stopped with emery or by a cork which has been long kept in melted paraffine. This cotton has been found very useful applied to glandular swellings of the neck. or wherever the local use of iodine is indicated—placing it around the neck, for example, and securing it by a bandage. It requires to be renewed every two or three days —*Med. Times and Gaz.*

---

*Treatment of Cancrum Oris.*—Of all the local remedies or applications I have resorted to in such cases, I have never found any application so useful or so effectual as hydrochloric acid. Neither nitric acid, nitrate of silver, nor chlorate of potash, nor any other remedy that I have ever tried or used, except hydrochloric acid, did I ever find to be of the least use to check cancrum oris. I have almost never found hydrochloric acid to fail to check the progress of this dreadful disease at once, and bring on a most rapid and healthy action in the part. Nor does it cause so much pain or suffering to the little patient as one would suppose, seeing that the gangrenous spot is almost entirely without feeling at this time. This acid is easily applied to the ulcer by means of a feather or small camel-hair brush. I have cured many cases of cancrum oris by this means.—*Dr. McGreevy in British Medical Journal.*

---

*Antidote to Carbolic Acid.*—Dr. T. Hasemann, from numerous careful experiments, both chemical and medicinal, advocates the use of a strong solution of saccharate of lime. of course to be taken as soon as possible, as an antidote to carbolic acid.—*Medical News.*

